# Medical Jurisprudence of Insanity

**Published:** 1876-03

**Authors:** 


### Books Reviewed.

---

*The Medical Jurisprudence of Insanity.* By J. H. BALFOUR BROWNE, Esq. Second Edition. With references to the Scotch and American Decisions. Philadelphia: Lindsay & Blakiston. 1876. Buffalo : T. Butler & Son.

There are few topics in medicine or jurisprudence which are more important than those connected with insanity. The responsibility of the insane for acts of crime, is a question which has attracted much attention from the professions of law and medicine, and received more notice from the secular press than any other. Scarcely is a crime of any great magnitude committed, especially if it is particularly atrocious in its details, before there are numerous persons who set up the plea of insanity in behalf of the supposed criminal.

The author of the work under consideration has had unusual facilities, for a non-professional man of observing the insane in the hospitals of Great Britain, and his work, therefore, will be found to be remarkably in accordance with the opinions entertained by medical men.

The author recognizes several of those forms of ansanity which have been the subject of doubt by some and of ridicule by others, among which are dipsomania, homocidal and suicidal mania, pyromania, kleptomania and erotomania.

The capacity of the insane is well discussed in the relation of making wills, executing contracts, etc.

The work is an interesting one, and the cases quoted and the decisions cited are instructive illustrations of the points taken by the author.

The publishers have presented the work in a very attractive and convenient form. We predict that it will find in its present form increased favor both in the professions of medicine and law.

